# Improving Diabetes Care in Rural Areas: A Systematic Review and Meta-Analysis of Quality Improvement Interventions in OECD Countries

**DOI:** 10.1371/journal.pone.0084464

**Published:** 2013-12-19

**Authors:** Ignacio Ricci-Cabello, Isabel Ruiz-Perez, Antonio Rojas-García, Guadalupe Pastor, Daniela C. Gonçalves

**Affiliations:** 1 Department of Primary Care Health Sciences, University of Oxford, Oxford, United Kingdom; 2 CIBER en Epidemiologia y Salud Pública (CIBERESP), Barcelona, Spain; 3 Andalusian School of Public Health, Granada, Spain; Nanjing Medical University, China

## Abstract

**Background and Aims:**

Despite well documented disparities in health and healthcare in rural communities, evidence in relation to quality improvement (QI) interventions in those settings is still lacking. The main goals of this work were to assess the effectiveness of QI strategies designed to improve diabetes care in rural areas, and identify characteristics associated with greater success.

**Methods:**

We conducted a systematic review and meta-analysis. Systematic electronic searches were conducted in MEDLINE, EMBASE, CINAHL, and 12 additional bibliographic sources. Experimental studies carried out in the OECD member countries assessing the effectiveness of QI interventions aiming to improve diabetes care in rural areas were included. The effect of the interventions and their impact on glycated hemoglobin was pooled using a random-effects meta-analysis.

**Results:**

Twenty-six studies assessing the effectiveness of twenty QI interventions were included. Interventions targeted patients (45%), clinicians (5%), the health system (15%), or several targets (35%), and consisted of the implementation of one or multiple QI strategies. Most of the interventions produced a positive impact on processes of care or diabetes self-management, but a lower effect on health outcomes was observed. Interventions with multiple strategies and targeting the health system and/or clinicians were more likely to be effective. Six QI interventions were included in the meta-analysis (1,496 patients), which showed a significant reduction in overall glycated hemoglobin of 0.41 points from baseline in those patients receiving the interventions (95% CI -0.75% to -0.07%).

**Conclusions:**

This work identified several characteristics associated with successful interventions to improve the quality of diabetes care in rural areas. Efforts to improve diabetes care in rural communities should focus on interventions with multiple strategies targeted at clinicians and/or the health system, rather than on traditional patient-oriented interventions.

## Introduction

Diabetes mellitus type 2 (DM2) is fast becoming the epidemic of the 21st century, being currently listed as the ninth cause of death worldwide [1]. In 2012 its global prevalence was 8.3%, affecting 371 million people, and it is estimated that by 2030 almost one tenth of the population will have DM2 (552 million people) [2].

Recent reviews have shown that quality of care for diabetes in the US [3], Europe [4], and Latin America [5] remains suboptimal, highlighting the existence of important disparities in the provision of diabetes care, with considerable gaps in the care provided to different population groups [6]. Although socioeconomic and ethnic disparities have been more frequently reported [7,8], several studies have also analyzed urban-rural disparities in access to and quality of DM2 care [9,10]. Evidence have already been found for disparities in healthcare provision in rural areas, with worsened health outcomes including poorer glycemic control, worse lipid profiles, and higher blood pressure [11]. 

Those disparities seem to be mainly explained by the lack of an infrastructure capable of sustaining the processes required to improve care and outcomes. Individuals living in rural communities often have to travel longer distances to obtain appropriate healthcare, which has been strongly associated with poorer glycemic control [12,13]. Access to diabetes specialists is scarcer in rural centers [14], and general practitioners are less likely to adhere to standards of DM2 care [15,16]. Furthermore, the lack of a multidisciplinary team increases the burden on the healthcare provider, who with limited time and resources has to both educate and treat the patient [17], often with poor clinical information systems or preventive health services and isolated from diabetes education programs [18]. 

The interplay between worsened health status and socioeconomic and cultural factors in rural patients with DM2 has also been explored. Results have indicated that several of those factors are associated with exacerbated barriers in adequate diabetes self-management (DSM), including financial strains, cultural barriers [19], mistrust, communication issues, and high rates of health illiteracy [20-22].

Quality improvement (QI) is a multidisciplinary, systems-focused, data-driven method of understanding and improving the efficiency, effectiveness, and reliability of health processes and outcomes of care [23]. Different types of QI interventions to improve diabetes care have been developed and assessed, and their potential to promote health outcomes has been ascertained [24,25]. Nonetheless, despite well documented disparities in health and healthcare in rural communities, there is a lack of evidence in relation to QI interventions in those settings. Given the potential for QI efforts to improve outcomes in the general population, considering whether these interventions could also be fruitful in rural settings is an essential next step. Therefore, the main goals of this systematic review were to assess the effectiveness of QI strategies designed to improve diabetes care in rural areas, and identify characteristics associated with greater success.

## Methods

The present work is part of a broader systematic review aimed to identify and analyze healthcare interventions to improve diabetes care in socially vulnerable population groups. The review and its procedures were planned, conducted, and reported according to the Preferred Reporting Items for Systematic Reviews and Meta-Analyses (PRISMA) guidelines [26].

### Data Sources and Searches

A comprehensive core search strategy was developed for Medline through Ovid (combining MeSH terms and keywords) and then adapted and implemented in EMBASE, and CINAHL (search strategy available in Table S1). Gray literature and additional articles were searched in twelve more bibliographic sources (search registry available in Table S2). The search was not restricted by language or publication date. For all the studies selected from this stage, backward and forward citation searches were performed in ISI Web of Knowledge. All searches were conducted from inception to October 2012. A bibliographical database was created using EndNote X6 and used to store and manage the retrieved references.

### Study Selection

**Figure 1:**
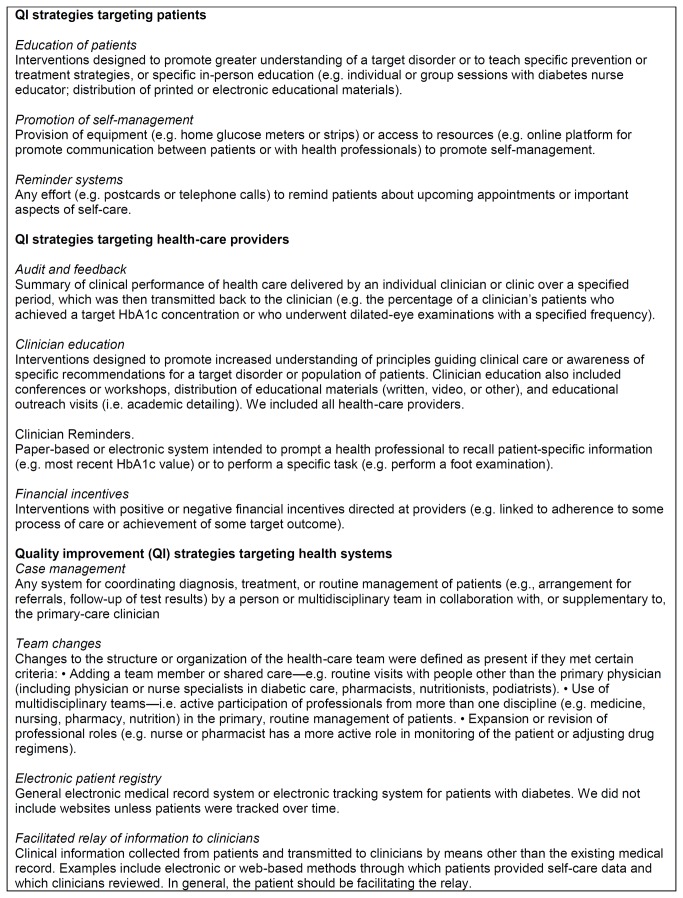
Taxonomy of quality improvement strategies (adapted from Shojania et al. [25] and Tricco et al [24]).

We included studies assessing the effectiveness of QI interventions to improve diabetes care in rural areas. For this study, we defined a QI intervention as an intervention directed towards healthcare systems, healthcare providers, or patients, for the purpose of increasing the likelihood of optimal clinical quality of care, measured in terms of processes of care, DSM, and clinical status. Specifically, we included those interventions meeting the definition of at least one out of 11 specific types of QI strategies, based on a taxonomy adapted from Shojania et. al. [25] (Figure 1). Interventions were eligible when targeting either professionals providing healthcare to patients with DM2 or patients with DM2, as defined by the studies.

Eligible designs were randomized controlled trials, including cluster randomized controlled trials; controlled trials, including quasi-randomized trials; controlled before-after studies; and non-controlled before-after studies. Studies including a control group were only eligible in those cases in which they received usual care. 

In order to ensure relevance to the different health systems in different countries we broadly defined rural areas as those geographic areas that are located outside cities and towns. 

To increase homogeneity among interventions we only included those interventions exclusively addressing DM2. For the same reason we restricted our review to those interventions which were conducted in OECD countries when study selection was started [27] (November 2012). Pilot studies for which the complete results have been published later were excluded.

Titles and abstracts were screened for eligibility, and those fulfilling the inclusion criteria were included in the next stage, where the full texts of the selected abstracts were retrieved and assessed. Those that met the inclusion criteria were included for data extraction. Two reviewers independently screened citations and full texts, and any disagreements were solved by a third reviewer. 

### Data Extraction and Quality Assessment

We designed and used structured forms to extract pertinent information from each article, including information about the methods and population characteristics, interventions, comparators, outcomes, timing, settings, and study design. 

The methodological quality of the studies was assessed using the “Quality Assessment Tool for Quantitative Studies” [28], which evaluates both internal and external validity. The methodological quality of each article was classified according to three categories (good, fair or poor), based on six aspects: selection bias, study design, confounders, blinding, data collection, and withdrawals and dropouts. Two independent reviewers extracted the information and conducted the quality assessment. Disagreements were resolved by consulting a third reviewer.

### Data Synthesis and Analysis

Interventions were classified both according to the targeted healthcare group (patients, clinicians, health system or multi-target interventions) and to the strategies that were delivered, following the taxonomy proposed by Shojania et. al. [25].

The effectiveness of the interventions was assessed based on its impact on processes of care, DSM, and clinical status. Processes of care were ascertained by variables reflecting either clinical tasks (number of HbA_1c_ tests in the past year and pneumococcal vaccination ever received, among others) or patient satisfaction with the healthcare received. DSM mainly included the performance of specific activities (diet, exercise, glucose control, foot self-examination), but also considered other aspects such as diabetes knowledge or confidence and skills to control diabetes. Finally, clinical status included all clinical measures normally used to monitor diabetes (HbA1c, body mass index, blood pressure, amongst others). For each study all outcomes used to assess the effectiveness of interventions were examined and, if applicable, classified as measuring one of these three domains. Variables that did not measure any of these three domains were not considered in the analysis.

Following a similar approach developed by our team in a previous review [29], the effectiveness of each intervention was rated in terms of each of the above mentioned domains as being “high”, “partial” or “low”, according to the percentage of variables in which the experimental group improved significantly after the intervention. Accordingly, the following categories of effectiveness were established: 1) highly effective interventions, for those interventions that showed statistically significant improvements in 75% or more of the measures; 2) partially effective interventions, for improvements between 25% and 75%; and 3) low-effectiveness interventions, when improvement was observed in less than one quarter of the measures. Chi-square test was used in order to assess differences in the effectiveness between types of interventions. A sensitivity analysis, excluding the studies with higher risk of bias, was also conducted.

Eligibility criteria for the meta-analysis included RCTs comparing the intervention with usual care and reporting HbA1c in the control and experimental group before and after the completion of the intervention. The mean (standard deviation) HbA1c levels in each study were extracted. This information was transformed into weighted mean difference, and 95% confidence intervals (CI) were calculated for all eligible studies in the meta-analysis, and combined using inverse-variance weighted random-effects models. Heterogeneity was quantified by the I^2^ statistic, where I^2^ ≥50% was considered evidence of substantial heterogeneity [30]. Sources of heterogeneity were investigated by a Galbraith plot. Publication bias was quantitatively assessed with Egger’s test. A meta-regression analysis was also performed to assess the possible effects of characteristics deemed potentially relevant, namely the healthcare group targeted the intervention, the duration or the number of strategies included. 

All analyses were conducted with Stata, version 12.0. For all the analyses, statistical significance was accepted at *p* < 0.05. 

## Results

### Article identification

**Figure 2:**
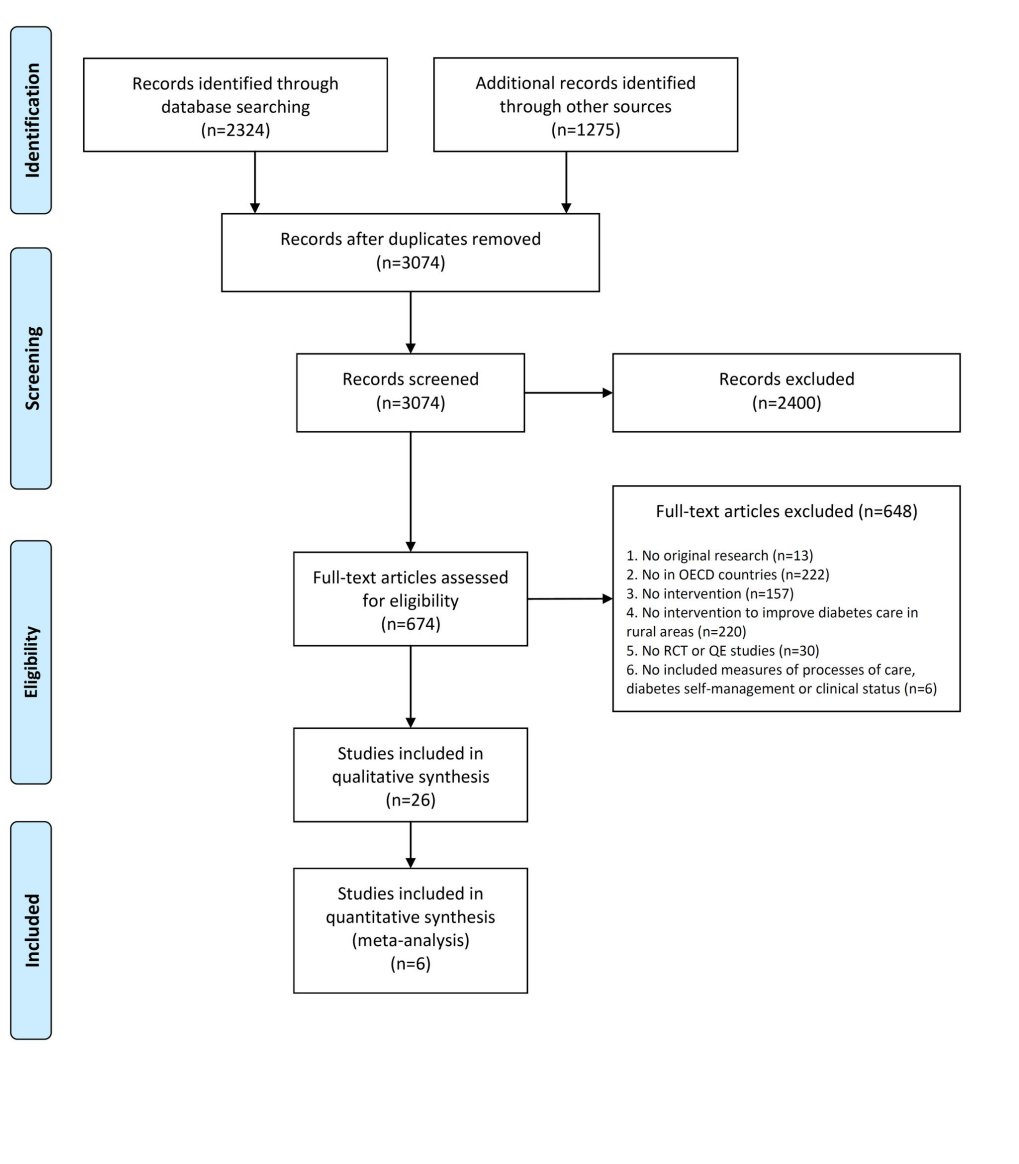
Summary of evidence search and selection. * Information regarding the sources is available on Table S2.

Search results are summarized in the PRISMA flow diagram (Figure 2). The initial search identified a total of 1,916 citations, 248 of which were duplicated. Title and abstract screening of the remaining 1,668 citations resulted in the identification of 465 citations for further review. After examination of full text articles, 21 articles were selected as being eligible. The search of backward and forward citations of these 21 articles retrieved five additional articles, resulting in 26 articles included [31-56], which reported 20 different interventions.

The discrepancy between the number of studies and interventions included can be explained by the reporting of more than one intervention per study, as well as the publication of more than one article for some of the studies. 

Specifically, sixteen different articles [31-39,41,43,44,48,49,52,53] reported on sixteen different interventions (one article per intervention). However, one article [47] reported a study about two different interventions, whereas three different articles [42,45,46] reported on the same intervention (the Diabetes Outreach Van Enhancement (DOVE) intervention), and six articles [40,50,51,54-56] reported on another intervention (The Informatics for Diabetes Education and Telemedicine (IDEATel) intervention). 

### Characteristics of the studies

Characteristics of the studies are summarized in Table 1 and fully described in Tables S3, S4, S5, and S6. The first article on this subject was published in 2000, and most of the following articles have been published from 2004 onwards. Fourteen of the twenty-six studies identified adopted a quasi-experimental design [32-35,39,41-46,48,49,52], whereas twelve were randomized clinical trials [31,36-38,40,47,50,51,53-56]. The number of participants ranged from 15 to 1,665, although almost one fourth of the studies did not exceed 40 participants. Only two studies included a follow up after the completion of the intervention to test their long term effects [42,53]. Regarding their methodological quality, six studies were assessed as having a low risk of bias [38,45,50,51,54,55], nine a medium risk [31,36,37,40,42,43,46,47,56], and 11 presented high risk [32,34,41,44,49,52,53]. The main methodological issues observed in studies with higher risk of bias were lack of blinding, possible selection bias, and high dropout rates. 

**Table 1:** Summary of characteristics and effectiveness of the interventions.

	**Number (N)**	**Percentage (%)**
**Country in which the intervention was conducted**		
US	18	90
Canada	1	5
Japan	1	5
**Setting**		
Primary Care center	16	80
Hospital	1	5
**Community center**	3	15
**Duration (months)**	3-38*	12.8 (9.1) †
**Type of interventions**		
Targeted to patients	9	45
Targeted to health providers	1	5
Targeted to the health system	3	15
Multiple targets	7	35
**Type of quality improvement strategies**		
*Targeted to patients*	16	80
Education of patients	16	80
Promotion of self-management	3	15
Reminder systems	2	10
*Targeted to healthcare providers*	4	20
Audit and feedback	1	5
Clinician education	4	20
Clinician reminders	0	0
Financial incentives	0	0
*Health system*	8	40
Case management	5	25
Team changes	2	10
Electronic patient registry	5	25
Facilitated relay of information to clinicians	1	5
**Number of quality improvement strategies**		
1	10	50
2	6	30
≥3	4	20
**Impact on clinical status**		
High	2	12.5
Partial	11	68.8
Low	3	18.8
Not analyzed	4	-
**Impact on diabetes self-management**		
High	5	45.5
Partial	6	54.5
Low	0	0
Not analyzed	9	-
**Impact on clinical processes of care**		
High	4	57.1
Partial	3	42.9
Low	0	0
Not analyzed	13	-

* Duration of the intervention expressed as minimum and maximum

^†^ Duration of the intervention expressed as mean (standard deviation)

### Characteristics of the interventions

Characteristics of the 20 interventions are summarized in table 1 and fully described in Table S3, Table S4, Table S5, and Table S6. Most of the interventions (90%) were conducted in the United States and took place in primary care settings (80%). Their duration ranged from 3 months to 5 years (mean=15.6-months, standard deviation=13.6). Nine different QI strategies were identified: three targeting patients (patient education, promotion of DSM, and reminder systems), two targeting healthcare providers (audit and feedback and clinician education), and four targeting the health system (case management, team changes, electronic patient registry, and facilitated rely of information to clinicians). No intervention included strategies such as clinician reminders or supplying financial incentives to healthcare providers. Half of the interventions included a single QI strategy, 30% included two strategies, and 20% three or more strategies. The vast majority of the interventions included at least one strategy targeted to patients, normally patient education (80%), one fifth included at least one strategy targeted to providers, and 40% included a strategy towards health system improvement.

Nine interventions exclusively targeted patients, all of which included educational strategies. In addition to patient education, two interventions also included promotion of DSM strategies, which consisted on the provision of equipment (e.g., home glucose meters) [33] or access to resources (e.g., system for electronically transmitting home glucose measurements) to promote DSM [53]. One intervention was exclusively targeted to clinicians, and consisted in providing training to small groups of GPs in a rural area in Alberta (Canada) [42,45,46]. Three interventions exclusively included QIs targeting the healthcare system. Of those, two consisted in the implementation of an electronic patient registry [41,49], whereas in the third a telemedicine system was installed in patients’ homes to facilitate interaction with health professionals and remote monitoring [40,50,51,54-56].

Seven interventions were directed towards more than one target. All of them included strategies targeting patients, most frequently patient education, although other also included promotion of DSM [43] or reminder systems for patients [35,36]. Clinician education and audit and feedback were included in one intervention [43]. Strategies towards the health system were also included in some interventions, consisting in the implementation of case management [31,32,35,36], electronic patient registries [35,36,39], and team changes [39].

### Effectiveness of the interventions

Out of the 20 interventions identified in this systematic review, seven were assessed in terms of their impact on processes of care, eleven on DSM, and sixteen on clinical status. The most positive effects of the interventions were observed in processes of care, with more than half of the studies assessing outcomes related with the processes of care being highly effective (combined sample of 1,376 participants). Five out of the eleven interventions that analyzed their effect on DSM (total N= 2,027 participants) were highly effective. Finally, only two out of the sixteen interventions that assessed the impact upon patients’ clinical status (total N= 1,878 participants) were highly effective. Sensitivity analysis excluding those studies with higher risk of bias showed similar results.

**Figure 3:**
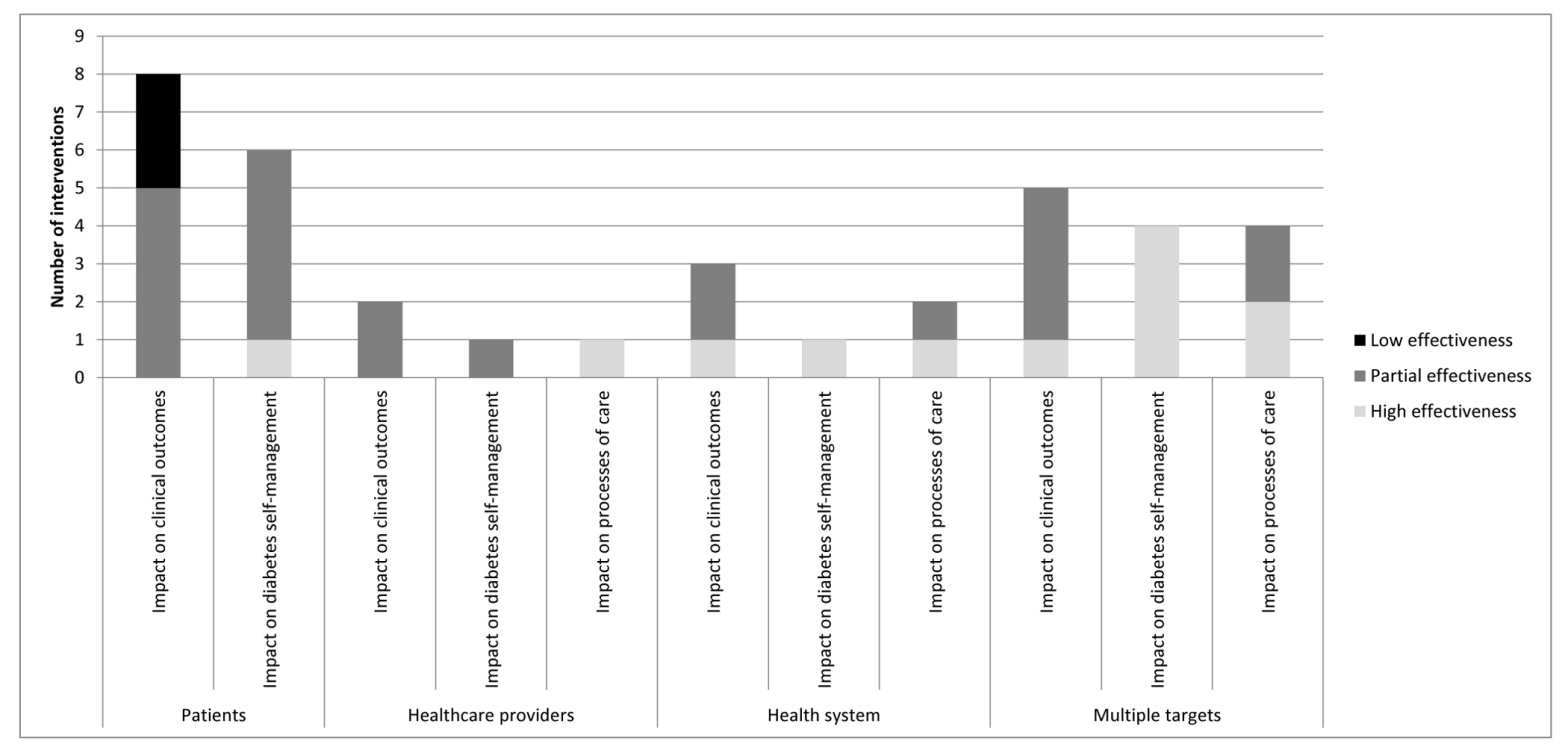
Effectiveness of quality Improvement interventions, by type of target.

Figure 3 displays the effectiveness of the interventions by type of target and outcome measure. None of the interventions targeting only patients was highly effective in improving DSM or clinical status. The only intervention that was exclusively targeted to clinicians produced a high impact on the clinical process of care, and partial effects in DSM and patients´ clinical status. Interventions targeting health system reported a high impact on DSM and processes of care, although only one out of four had a high impact on clinical status. All multi-target interventions assessed in terms of their impact on DSM reported a high impact. Additionally, half were highly effective in improving processes of care, although only one out of five showed the same result in clinical status.

The number of strategies employed per intervention was also associated with their effectiveness, with those interventions including a higher number of strategies producing a greater impact on DSM and clinical status. Thus, only about a fifth of the interventions including a single strategy produced a high impact in the improvement of DSM, whereas this percentage increased to 80% in interventions including two strategies and to all of those including three or more (statistically significant difference at *p*<0.05). Although not statistically significant, a similar trend was observed in relation to clinical outcomes: less than one tenth of those including a single strategy were highly effective, compared with one fifth of those including two, and half of those including three or more strategies.

**Figure 4:**
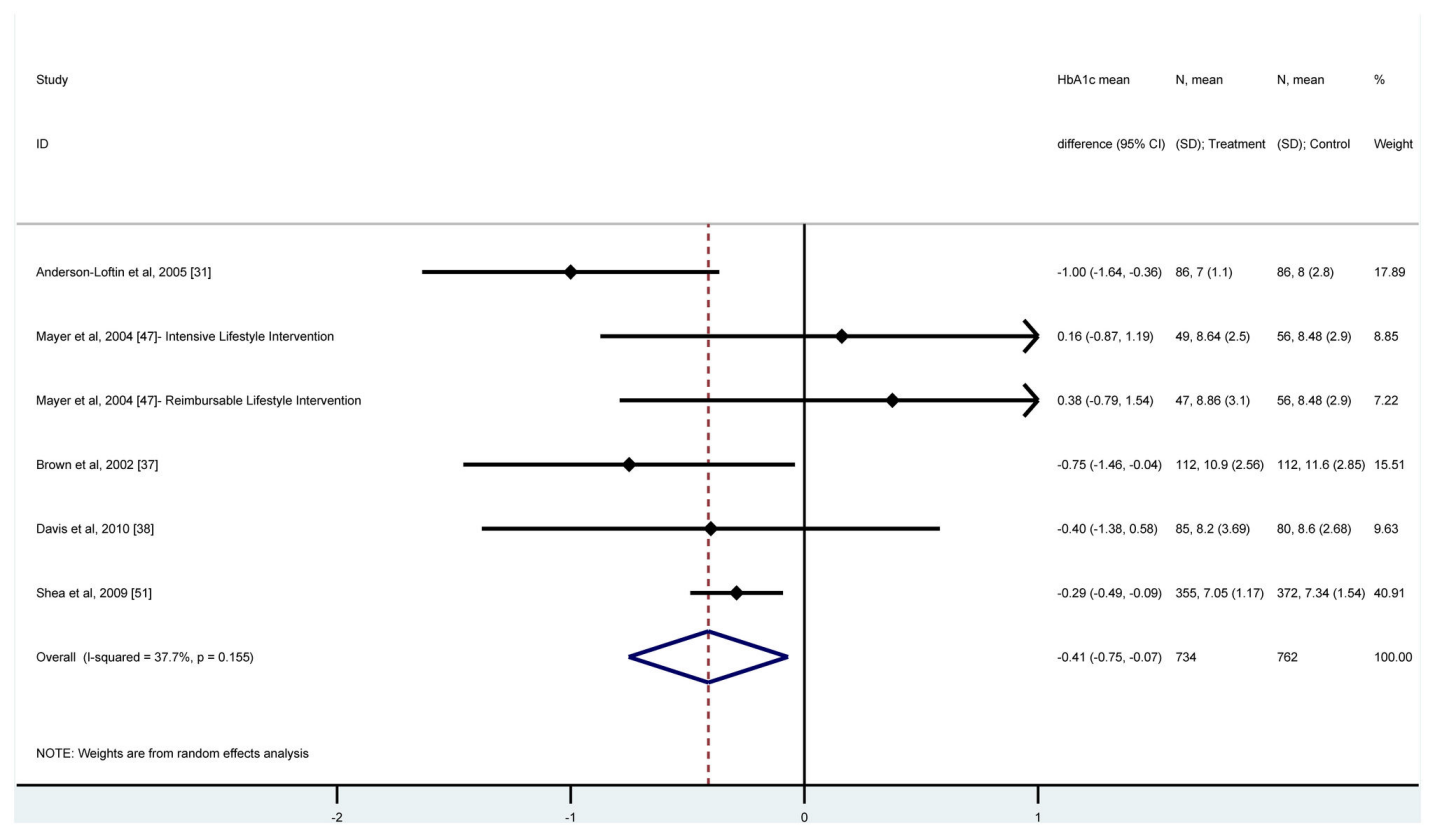
Meta-analysis of the effect of quality improvement interventions on glycated hemoglobin. Hba1c=Glycated hemoglobin. N=number of participants. SD=standard deviation. I squared=Variation in standardized mean difference attributable to heterogeneity.

Seven interventions were initially included in the meta-analysis [31,36-38,47,51]. The remaining interventions were excluded as they employed a study design other than RCT [32-35,39,41-46,48,49,52] or did not measure HbA1c [53]. A first meta-analysis was conducted to assess possible baseline HbA1c differences between intervention and control groups, observing no statistically significant differences (HbA_1c_ mean difference = -0.065% [95% CI -0.31% to 0.18%]). Subsequently, a second meta-analysis was conducted in order to estimate the pooled difference in HbA_1c_ between the intervention and control group after the intervention was implemented, which showed a substantially high heterogeneity (I^2^=61.6%). By using a Galbraith plot we identified the intervention published by Bray et al. [36] as the main source of heterogeneity. Removing this intervention from the meta-analysis substantially decreased the heterogeneity (I^2^= 37.7%). Figure 4 illustrates the result of the final meta-analysis, which included six interventions and 1,496 participants (734 in the intervention and 762 in the control group), observing that the combined effect of the intervention produced a significant reduction in the overall HbA_1c_ of 0.41% (95% CI -0.75% to -0.07%). Egger’s test showed an absence of publication bias (*p*=0.84). 

Although no statistically significance differences were observed, meta-regression indicated that several characteristics increased the effectiveness of the interventions (table 2). Thus, those interventions addressing multiple targets, including multiple QI strategies, employing case management as a QI strategy, delivered in community instead of clinic settings, and/or not requiring participants to commute to receive the intervention decreased HbA1c more effectively than the rest of interventions.

**Table 2:** Meta-regression of the effect of intervention´s characteristics on pooled glycated hemoglobin.

**Intervention characteristics**	**Number Interventions**	**SMD**	**95% CI**	**Residual I^2^**
All interventions	6	-0.41	-0.75 to -0.07	37.7%
**Targets**				17.48%
Patients	4	Ref.	-	-
Providers	0	-	-	
Health System	1	-0.02	-1.27 to 1.23	
Patients and Health system	1	-0.73	-2.32 to 0.60	
**Duration**				17.48%
3 months	1	Ref.	-	-
6 months	1	-0.71	-2.31 to 0.90	
12 months	4	0.02	-1.23 to 1.27	
**Setting**				42.88%
Primary Care	4	Ref.	-	-
Community	2	-0.28	-1.51 to 0.96	
**Patient Education**				39.00%
No	1	Ref.	-	-
Yes	5	-0.17	-1.42 to 1.08	
**Case Management**				50.01%
No	4	Ref.	-	-
Yes	2	-0.31	-1.49 to 0.88	
**Facilitated relay of information to clinicians**				39.00%
No	5	Ref.	-	-
Yes	1	0.17	-1.08 to 1.42	
**Number of QI strategies**				50.01%
1	4	Ref.	-	-
2	2	-0.31	-1.49 to 0.87	
**Intervention involving patients commuting**				21.12%
Yes	4	Ref.	-	-
No	2	0.41	-0.46 to 1.28	

SMD: standardized mean difference; I^2^: Variation in standardized mean difference attributable to heterogeneity; QI: Quality improvement

## Discussion

The present study has identified 20 healthcare-led interventions specifically aimed at improving the quality of DM2 care in rural areas. These interventions targeted patients, clinicians or the health system, sometimes including more than one target, and implemented one or more QI strategies. The most common type of interventions were those exclusively targeting patients, and the most common strategy was patient education, which was implemented either as a single strategy or in tandem with other strategies. Interventions mainly improved processes of care and DSM, and lesser impact was observed for health outcomes. Interventions including strategies targeting the health system and/or clinicians produced stronger positive outcomes than those targeting only patients. Also, interventions including multiple QI strategies were more effective than those that only included one.

Previous systematic reviews analyzed the effect of QI strategies on diabetes care in the general population [24,25], ethnic minorities or socially disadvantage populations in general, observing positive impact upon health outcomes [29,57-59]. However, this is the first systematic review that has purposely analyzed the impact of QI interventions on DM2 healthcare provided to patients in rural communities. In this systematic review we have observed that interventions are more likely to produce a positive effect on processes of care and DSM, but less frequently caused a high impact on health outcomes, which should be the final goal of these interventions. If we think in the organization of healthcare delivery as conceptualized by Donabedian [60], improvements in structure and processes should lead to improvement in patients’ health. The observed lack of positive impact on health could be at least partially attributed to the short length of the interventions, which lasted on average one year. This period might be enough to produce a positive effect on processes and DSM, but not for triggering an observable impact on health outcomes.

Most of the interventions targeted patients, with a smaller percentage of interventions with strategies targeting the health system. This is surprising, not only when considering that one of the main barriers for healthcare provision in rural areas is the lack of infrastructures for supporting care processes, but also that interventions exclusively targeting patients produced the lowest effect, which has been corroborated by this systematic review. The meta-analysis further reiterated those results, which showed that multi-target interventions significantly improve glycemic control, but not patient targeted interventions.

A dose response effect was also observed in relation to the number of strategies used in each intervention, both in relation to DSM and health outcomes. The absence of an effect for processes of care might be explained by the low number of interventions with multiple strategies found for that type of outcomes. Similar results were found in a previous systematic review of interventions for African-American patients [29], and the need to intervene at multiple levels with multiple strategies has been already highlighted [59].

Similarly, it has been observed that interventions directed toward organizational structures and resources, which went beyond traditional diabetes education programs, were more likely to be successful in socially disadvantaged populations [57].

### Strengths and weaknesses

The main strength of this study is the comprehensiveness of the searches. Systematic and manual searches were performed in the most relevant bibliographic databases on biomedical research, as well as in specific sites of gray literature. Also, the impact of the interventions on processes of care, DSM, and health outcomes was independently assessed, which allowed a deeper understanding of the effect of the interventions. 

Our review also has some limitations. First, although the scope of the review was OECD countries, a vast majority of the interventions found were conducted in US, which limits the external validity of our study. Second, we rated the effectiveness of each intervention according to the percentage of variables that significantly improved after the intervention. This approach does not take into account the clinical significance of this improvement or the fact that some outcome measures might be considered more relevant than others. Third, there were considerable differences in the characteristics of the studies assessing the main types of interventions: interventions targeted to patients were more frequently assessed through RCTs and included a lower number of participants, whereas the remaining interventions were more frequently assessed by before-after studies and included a higher number of participants. These differences could have impacted the internal validity of the results. Additionally, our meta-analysis and meta-regression were restricted to glycemic control. We agree that glycemic control is only one of several important outcomes for patients with diabetes and that the use of other outcomes such a fasting blood glucose, cholesterol levels or body mass index could also provide additional and relevant information. However, these outcomes were not uniformly available and therefore it was not possible to include them in our meta-analytic approach. Finally, although Egger´s test suggested the absence of publication bias, we cannot completely rule out its existence because of the low number of interventions included in the meta-analysis.

### Remaining Gaps in Knowledge

This review identified a lack of published studies analyzing strategies to improve quality of care in rural areas from countries other than the US. It has also indicated the need of increasing the methodological quality of the studies conducted. This was particularly true for the studies analyzing the effectiveness of multi-target interventions, which were more frequently evaluated through non-controlled studies. Also, most of the interventions had a relatively short duration and no follow-up, which prevented us from knowing whether the positive effects observed on processes of care and DSM were maintained on the long-term, and whether a more positive effect on health outcomes can be observed if interventions were longer. Given the interest in “pay for performance” schemes during the last decade the lack of studies of this type of interventions in rural areas was surprising. These aspects constitute areas for future research. 

## Conclusions

Interventions to improve quality of diabetes care in rural areas are at least partially effective. Stronger positive outcomes are associated with interventions that 1) contain strategies targeting providers or health system level and 2) simultaneously implement multiple strategies. Finally, the majority of the interventions designed to improve diabetes care in rural areas have mainly focused on patient education. However, these interventions seem to be the least effective option.

## Supporting Information

Table S1 **Search strategy in Medline (Ovid).**
(DOCX)

Table S2 **Registry of the Bibliographic Searches.**
(DOCX)

Table S3 **Characteristics and effectiveness of the quality improvement interventions targeted to patients.** QI= quality improvement; N= number of participants; CO = clinical outcomes; DSM = diabetes self-management; RCT = randomized, controlled trial; BMI = body mass index; QE = quasi-experimental study; LDL-c= low-density lipoprotein cholesterol; NA= not analyzed; SMBG= self-monitoring of blood glucose; HbA1c= glycated hemoglobin; DM2= type 2 diabetes mellitus.
*. Outcomes measures which showed a statistically significant improvement after the intervention are marked bold.
(DOCX)

Table S4 **Characteristics and effectiveness of the interventions targeted to health providers.** QI= quality improvement; N= number of participants; CO = clinical outcomes; DSM = diabetes self-management; PC= processes of care; QE = quasi-experimental study; NA= not analyzed; HbA1c= glycated hemoglobin .
*. Outcomes measures which showed a statistically significant improvement after the intervention are marked bold.
(DOCX)

Table S5 **Characteristics and effectiveness of the interventions targeted to health system.** QI= quality improvement; N= number of participants; CO = clinical outcomes; DSM = diabetes self-management; PC= processes of care; DM2= type 2 diabetes mellitus; RCT = randomized, controlled trial; BMI = body mass index; QE = quasi-experimental study; LDL-c= low-density lipoprotein cholesterol; HDL-c = high-density lipoprotein cholesterol; NA= not analyzed; HbA1c= glycated hemoglobin.
*. Outcomes measures which showed a statistically significant improvement after the intervention are marked bold.
(DOCX)

Table S6 **Characteristics and effectiveness of multi-target interventions.** QI= quality improvement; N= number of participants; CO = clinical outcomes; DSM = diabetes self-management; PC= Processes of care; DM2= type 2 diabetes mellitus; RCT = randomized, controlled trial; BMI = body mass index; QE = quasi-experimental study; LDL-c= low-density lipoprotein cholesterol; HDL-c = high-density lipoprotein cholesterol; NA= not analyzed; HbA1c= glycated hemoglobin; SMBG= self-monitoring blood glucose.
*. Outcomes measures which showed a statistically significant improvement after the intervention are marked bold.
(DOCX)

Checklist S1 **Prisma Checklist.**
(DOC)
