# Supplementary material for: Improving Diabetes Care in Rural Areas: A Systematic Review and Meta-Analysis of Quality Improvement Interventions in OECD Countries
Source: PLoS One. 2013 Dec 19;8(12):e84464. doi: 10.1371/journal.pone.0084464 (PMC3868600; doi:10.1371/journal.pone.0084464)
Supplement: Table S1 — Search strategy in Medline (Ovid). (DOCX) [file pone.0084464.s001.docx]

Table S1. Search strategy in Medline (Ovid)

| **Nº** | **Search** |
| --- | --- |
| 1 | exp *Healthcare Disparities/ or *Spouse Abuse/ or *Battered Women/ or exp *Health Status Disparities/ or exp *Poverty Areas/ or exp *Poverty/ or *Vulnerable Populations/ or Medical Indigency/ or *Rural health/ |
| 2 | exp Healthcare Disparities/ or exp Health Status Disparities/ or exp Poverty Areas/ or exp Poverty/ or Vulnerable Populations/ or *Spouse Abuse/ or Battered Women/ or Rural health/ |
| 3 | ("in need" or poverty or gender-bias or low-income or underserved or inequit$ or inequalit$ or disadvantag$ or disparit$ or inner-city or rural).ti. |
| 4 | ("in need" or poverty or gender-bias or low-income or inequit$ or inequalit$ or disadvantag$ or disparit$ or inner-city).ti,ab. |
| 5 | exp Ethnic Groups/ or Cultural Diversity/ or Minority Health/ or Minority Groups/ |
| 6 | exp Sex Factors/ or exp Socioeconomic Factors/ or Gender Identity/ or *Women/ or Women, Working/ |
| 7 | exp Social Problems/ or exp Social Conditions/ or exp Social Environment/ or Social Distance/ or exp Social Isolation/ or exp Social Class/ |
| 8 | (minorit$ or immigrant$ or racial or ethnic$ or elder$ or women or gender or disab$ or ((social or psychosocial or educational) and (differences or problems or population$))).ti. |
| 9 | (2 or 4 or 7) and (5 or 6 or 8) |
| 10 | exp diabetes mellitus/ or diabetes.ti. |
| 11 | (1 or 3 or 10) and 11 |
| 12 | Community health services/ or health care rationing/ or Healthy People Program/ or exp capacity building/ or exp health facilities/ or exp health personnel/ or health promotion/ or exp *health services/ or health care reform/ or health plan implementation/ or health planning technical assistance/ or health priorities/ or health resources/ or national health programs/ or regional health planning/ or exp Preventive Health Services/ or health education/ |
| 13 | exp "analytical, diagnostic and therapeutic techniques and equipment (non mesh)"/og, ec, st, ut |
| 14 | health services accessibility/ |
| 15 | exp Self Care/ not Self Medication/ |
| 16 | (intervention$ or plan$ or education or adherence or monitor$ or strateg$ or provision or address or attenuat$ or servic$ or measur$ or program? or programme? or prevention or diagnost* or treatment or follow-up or promotion or care or self-care or reminder or manag$ or self-manag$ or provid$ or rehabilitat$ or improv$ or prevent or preventing or tackling or address$ or attenuat$ or reduc$ or ((favor or favour or promote) and (equit$ or fairness or equality))).ti. |
| 17 | 12 or 13 or 14 or 15 or 16 |
| 18 | 11 and 17 |
| 19 | (quasi-experimental or evaluat$ or evidence or assessment or effectiveness or 'health survey' or trial or cohort or "longitudinal study" or utilization or access$).ti. or (quasi-experimental or random$ or 'health survey' or "longitudinal study").ab. or ((cohort or comparative or control$ or prospective or evaluation or blind$ or effectiv$) adj2 (study or trial)).ti,ab. |
| 20 | exp cohort studies/ or exp clinical trials as topic/ or exp clinical trial/ or feasibility studies/ or intervention studies/ or comparative studies/ or evaluation studies/ or validation studies/ |
| 21 | (systematic adj2 (review$ or overview)).ti. or (REVIEW.pt. and (systematic adj1 (review$ or overview)).ab.) or exp meta-Analysis/ or (meta-anal$ or metaanal$ or meta anal$).ti,ab. |
| 22 | 19 or 20 or 21 |
| 23 | 18 and 22 |
| 24 | limit 18 to (systematic reviews or meta analysis) |
| 25 | 23 or 24 |
