# Supplementary material for: Improving Diabetes Care in Rural Areas: A Systematic Review and Meta-Analysis of Quality Improvement Interventions in OECD Countries
Source: PLoS One. 2013 Dec 19;8(12):e84464. doi: 10.1371/journal.pone.0084464 (PMC3868600; doi:10.1371/journal.pone.0084464)
Supplement: Table S2 — Registry of the Bibliographic Searches. (DOCX) [file pone.0084464.s002.docx]

Table S2. Registry of the Bibliographic Searches

| **Databases** | **Platform/ Access** | | **Search date** | | **References retrieved** | |
| --- | --- | --- | --- | --- | --- | --- |
| **Core databases** | | | | | | |
| EMBASE | Ovid Licensed Resource | | 27/10/2012 | | 770 | |
| Medline | Ovid Licensed Resource | | 27/10/2012 | | 689 | |
| CINAHL | EBSCO Licensed Resource | | 27/10/2012 | | 116 | |
| Current Contents | ISI Licensed Resource | | 27/10/2012 | | 355 | |
| CRD Databases (DARE, HTA) | [http://www.crd.york.ac.uk/crdweb/](http://nhscrd.york.ac.uk/) | | 27/10/2012 | | 6 | |
| **Coverage/regulatory/licensing agencies** | | | | | | |
| U.S. Centers for Medicare & Medicaid (CMS) Web site | [http://www.cms.hhs.gov/default.asp?](http://www.coverageandpayment.com/) | | 18/10/2012 | | 0 | |
| AETNA | <http://www.aetna.com/index.htm> | | 18/10/2012 | | 0 | |
| National Guidelines Clearinghouse | [http://www.ngc.gov](http://www.ngc.gov/) | | 18/10/2012 | | 0 | |
| Mc Master Health Forum | <http://www.mcmasterhealthforum.org/healthsystemsevidence-en> | | 20/10/2012 | | 3 | |
| **Grey literature** | | | | | | |
| Networked Digital Library of Theses and Dissertations - electronic theses and dissertations (ETDs) | <http://www.ndltd.org/> | | 20/10/2012 | | 0 | |
| **Other Internet Directories or search resources** | | | | | | |
| Health Evidence | <http://www.evidence.nhs.uk/> | | 20/10/2012 | | 5 | |
| SCIRUS | http://www.scirus.com/ | | 27/10/2012 | | 380 | |
| LILACS - Literatura Latinoamericana y del Caribe en Ciencias de la Salud | <http://bases.bireme.br/cgi-bin/wxislind.exe/iah/online/?IsisScript=iah/iah.xis&base=LILACS&lang=e> | | 25/10/2012 | | 0 | |
| CEA Registry | <https://research.tufts-nemc.org/cear/default.aspx> | | 25/10/2012 | | 0 | |
| metaRegister of Controlled Trials (mRCT) | http://www.controlled-trials.com/mrct/ | | 25/10/2012 | | 0 | |
| Backward and forward search of the articles previously identified. ISI WEB OF KNOWLEDGE | <http://apps.webofknowledge.com/> | | 30/11/2012 | | 1275 | |
| **TOTALREFERENCES SCREENED** |  |  | | **3599** | |  |
