# Supplementary material for: Improving Diabetes Care in Rural Areas: A Systematic Review and Meta-Analysis of Quality Improvement Interventions in OECD Countries
Source: PLoS One. 2013 Dec 19;8(12):e84464. doi: 10.1371/journal.pone.0084464 (PMC3868600; doi:10.1371/journal.pone.0084464)
Supplement: Table S3 — Characteristics and effectiveness of the quality improvement interventions targeted to patients. QI= quality improvement; N= number of participants; CO = clinical outcomes; DSM = diabetes self-management; RCT = randomized, controlled trial; BMI = body mass index; QE = quasi-experimental study; LDL-c= low-density lipoprotein cholesterol; NA= not analyzed; SMBG= self-monitoring of blood glucose; HbA1c= glycated hemoglobin; DM2= type 2 diabetes mellitus. *. Outcomes measures which showed a statistically significant improvement after the intervention are marked bold. (DOCX) [file pone.0084464.s003.docx]

Table S3. Characteristics and effectiveness of the quality improvement interventions targeted to patients

| **QI strategy / Duration (months)** | **Setting of intervention** | **Provider of intervention** | **Target population** | **Components of the intervention** | **Design/ N/ Follow-up** | **Analyzed variables and main results*** | **Overall quality** | **Impact on CO** | **Impact on DSM** | **Author (s)/ Country** |
| --- | --- | --- | --- | --- | --- | --- | --- | --- | --- | --- |
| Patient education + Promotion of self-management/5 | Rural health centers of Montana | Diabetes nurse educator | Women with diabetes between the ages of 35 and 60 years, living at least 25 miles outside the 6 major cities of Montana | Patients received a telemedicine unit with 4 functions: 1. Conversation: women talk among themselves about any topic with the purpose of creating a social support group (open conversations to all participants). 2. Private messages area: where women can talk among themselves or with the nurses. 3. Health area: special platform to address diabetes education. The nurse took part actively, raising questions about diabetes care. 4. Resources area: participants were provided with several resources (mainly educational) related to diabetes control. | RCT/ N=30 (intervention=15, control=15)/ Control group received usual care. Follow-up every 2.5 months during 10 months (final follow up at 5 months post-intervention). | DSM: Psychosocial adjustment to disease. | Poor | NA | Low | Smith & Weinert (2000)[53] / United States |
| Patient education /12 | Starr County, Texas-Mexico border | Bilingual Mexican American nurses, dietitians, and community workers. | Rural Mexican American with DM2, age between 35 and 75 years | Intervention consisted in 52 contact hours over 12 months. Involved 3 months of weekly instructional sessions on nutrition, SMBG, exercise, and other self-care topics and 6 months of biweekly support group sessions to promote behavior changes. The approach was culturally competent in terms of language, diet, social emphasis, family participation, and incorporation of cultural health beliefs. | RCT/ N=256 (intervention =128, control =128). Control group received usual care/ Follow-up at 3 and 6 months post-baseline and at completion. | CO: **HbA_1c_**, **fasting blood glucose**, cholesterol, triglycerides, BMI/ DSM: **diabetes knowledge**, health beliefs | Fair | Partial | Partial | Brown et al.[37] (2002)/ United States |
| Patient education /12 | Two primary health care centers in rural counties in South Carolina | Nutritionists | Ethnically diverse, medically underserved patients with DM2; age ≥ 45 years; BMI≥ 25 kg/m^2^ | **Intensive lifestyle intervention:** Educational intervention which had as main contents: low-calorie diets, physical activity and tools for self-control of both diet and physical activity. Participants met weekly with the study nutritionist for delivery of the first 4 months of the core curriculum (intensive), every other week for the next 2 months (transition), and once a month for the remaining 6 months (maintenance). Sessions were conducted sequentially in a pattern of 3 group sessions and 1 individual session. | RCT/ N=98 (Intervention=49, Control=49). Control group: usual care./ Follow-up at 3 and 6 months post-baseline and at completion. | CO: **BMI,** HbA_1c_, lipid profile and blood pressure. | Fair | Partial | NA | Mayer-Davis et al. (2004)[47] / United States |
|  |  |  |  | **Reimbursable-lifestyle intervention:** condensed version of the intensive-lifestyle intervention, in which key elements of the intensive-lifestyle intervention were delivered in 4 1-hour sessions over the course of the 12-month study and included 3 group sessions and 1 individual session. | RCT/ N=96 Intervention =47, Control=49). Control group: usual care/ Follow-up at 3 and 6 months post-baseline and at completion. | CO: BMI, HbA_1c_, lipid profile and blood pressure. | Fair | Low | NA | Mayer-Davis et al. (2004)[47] / United States |
| Patient education /12 | Primary care center in a rural area in Japan | Dietitians, exercise trainers and doctors | Elderly outpatients with DM2 who had seen a physician for 10 or more years in a Japanese rural area | Self-help group sessions (12 monthly sessions during 1 year) covering the following aspect of diabetes care: nutrition, exercise, diabetes complications, stress management and moral support. | QE (controlled before-after study)/ N= 15 (Intervention = 9, Control = 6)/ Follow-up at completion | DSM: **Diet behavior, Exercise behavior,** Diabetes knowledge | Poor | NA | Partial | Kotani et al. (2004) [44]/ Japan |
| Patient education /12 | 12 clinics in rural underserved counties in Arkansas | Registered nurse and dietitian | Patients with DM2 from hospitals or clinics in medically underserved rural areas | DSM education. After a 1-hour assessment of their educational needs, participants received 10 hours of diabetes education and 3 hours of medical nutrition therapy. Diabetes education was divided into three visits: an initial visit occurring shortly after the initial education assessment, a second at 6 months, and the third 1 year after program entry. | QE (non-controlled before-after study)/ N=319/ Follow-up at 6 months post-baseline and at completion | CO: Blood pressure, HbA1c/ DSM: SMBG, **foot self-examination** | Poor | Low | Partial | Balamurugan et al. (2006) [34]/ United States |
| Patient education + promotion of self-management/3 | Private hospital in Clinton, Arkansas | University of Arkansas together with the hospital | Patients with DM2 living in a medically underserved rural areas (mean age =67; average duration of diabetes =9 years). | **Patient education.** The program included 6 biweekly group sessions (two 1-hour sessions and four 2-hour sessions) conducted online. These sessions combined didactic presentation, demonstration, and interactive discussions. Sessions covered 9 content areas: diabetes disease process, nutrition, physical activity, medication, monitoring/using results, acute complications, chronic complications, goal setting and problem solving. **Promotion of self-management.** Glucose monitors and strips were provided to those participants who needed them. Weekly incentives were also provided. | QE (non-controlled before-after study)/ N=38/ Follow-up at completion | CO: HbA1c, cholesterol, blood pressure, triglycerides, microalbumin, weight/ DSM: SMBG, Foot self-examination**, Knowledge to manage diabetes**, **Skills to manage diabetes, Feel helpless to manage diabetes,** Can control diabetes myself, Confident to manage diabetes | Poor | Low | Partial | Balamurugan et al. (2009) [33]/ United States |
| Patient education + promotion of self-management/ 12 | 3 community health centers in northeast South Carolina | Nurse (Certified Diabetes Educators) and dietitian | Patients with DM2 from three community health centers in northeast South Carolina; HbA1c>7%; age >35 years; BMI > 25 kg/m^2^ | **Patient education.** DSM education intervention with 13 sessions, 3 individual and 10 groups. Two sessions (one individual and one group) were held in the first month for an intervention “jump start.” Three group sessions were conducted in-person. All others were conducted by interactive videoconferencing. **Promotion of self-management.** Intervention participants were offered retinal imaging in the primary-care setting when they were due for their annual eye exam. | RCT /N= 168 (intervention =85, control =80)/ Control group: usual care/ Follow-up at 6 months post-baseline and at completion | CO: **HbA_1c_, LDL-c**, Systolic blood pressure, Diastolic blood pressure, **BMI**, Waist circumference, Albumin-to-creatinine ratio | Good | Partial | NA | Davis et al. (2010)[38]/ United States |
| Patient education /6 | Rural Medical Clinics | Nurse Educator | Patients from two rural clinics with DM2; age≥18 years | Education sessions took place in the office or via telephone approximately every 6 weeks. The nurse educator provided in-person education in the office if the participant had a scheduled visit within the prescribed timeframe. Otherwise, she phoned the participant to provide the education. Contents included topics requested by the patients. | QE (controlled before-after study)/ N= 98 (Intervention = 48, Control = 50). Control group: usual care/ Follow-up at 3 months post-baseline and at completion. | CO: Weight, HbA_1c_, **Glucose/** DSM: **Diabetes Knowledge** eating habits, medication compliance, physical activity, feet check, **SMBG** | Poor | Partial | Partial | McIlhenny et al. (2011) [48]/ United States |
| Patient education /6 | Rural Medical Clinics | Nurse Educator | Patients from two rural clinics with DM2; age≥18 years | Each health clinic was supplied with a laptop computer for participants to use if they lacked internet access. Participants received verbal and/or written instructions given by the provider, an instructional handout that included step-by-step instructions how to access the My HERO web portal. In addition, intervention group received one on one healthcare education and hands-on instruction from a nurse educator on how to access and navigate My HERO. Education sessions took place in the office or via telephone approximately every 6 weeks. | QE (controlled before-after study)/N= 98/ (Intervention = 48, Control = 50)/ Control group (minimal intervention)/ Follow-up at 3 months post-baseline and at completion. | CO: Weight, HbA_1c_, **Glucose**/ DSM: eating habits, medication compliance, physical activity, feet check, **SMBG** | Poor | Partial | Partial | McIlhenny et al. (2011) [48]/ United States |
