# Supplementary material for: Improving Diabetes Care in Rural Areas: A Systematic Review and Meta-Analysis of Quality Improvement Interventions in OECD Countries
Source: PLoS One. 2013 Dec 19;8(12):e84464. doi: 10.1371/journal.pone.0084464 (PMC3868600; doi:10.1371/journal.pone.0084464)
Supplement: Table S4 — Characteristics and effectiveness of the interventions targeted to health providers. QI= quality improvement; N= number of participants; CO = clinical outcomes; DSM = diabetes self-management; PC= processes of care; QE = quasi-experimental study; NA= not analyzed; HbA1c= glycated hemoglobin . *. Outcomes measures which showed a statistically significant improvement after the intervention are marked bold. (DOCX) [file pone.0084464.s004.docx]

Table S4. Characteristics and effectiveness of the interventions targeted to health providers

| **QI strategy / Duration (months)** | **Setting of intervention** | **Provider of intervention** | **Target population** | **Components of the intervention** | **Design/ N/ Follow-up** | **Analyzed variables and main results*** | **Overall quality** | **Impact on CO** |  |  | |  | |  |
| --- | --- | --- | --- | --- | --- | --- | --- | --- | --- | --- | --- | --- | --- | --- |
|  |  |  |  |  |  |  |  |  | **Impact on DSM** | | **Impact on PC** | | **Author (s)/ Country** | |
| Clinician education /6 | Two comparable and geographically adjacent rural health regions in Northern Alberta | Group of specialists in diabetes (specialized physicians, nurse educators, dietitians, and pharmacists). | Primary care physicians | A team of experts in diabetes traveled monthly to the largest communities in the region during 6 months. Educational messages were delivered to small groups of primary care physicians. Specific components of the intervention included small group discussions of real and theoretical cases related to risk factors, delivered specialists, one-on-one academic detailing by a trained pharmacist, and a referral service for a limited number of patients. | QE (controlled before-after study)/ N=372 (intervention=200, control=172). Control group: minimal intervention/ Follow-up at completion | CO: HbA_1c_, blood pressure, and **total cholesterol** | Good | Partial | NA | | NA | | Majumdar et al. (2003)[46]/ Canada | |
|  |  |  |  |  |  | PC: **satisfaction with healthcare received /**DSM: Adherence to self-care activities, **diabetes self-efficacy, beliefs and attitudes** | Fair | NA | Partial | | High | | Maddigan et al. (2004)[45] / Canada | |
|  |  |  |  |  | QE (controlled before-after study)/ N=381 (200 original intervention group, 181 crosser-over intervention group). Control group: usual care / Follow up at 6 completion and at 9 months post-intervention | CO: Blood Pressure, **Cholesterol, HbA_1c_** | Fair | Partial | NA | | NA | | Johnson et al. (2005) [42]/ Canada | |
