# Supplementary material for: Improving Diabetes Care in Rural Areas: A Systematic Review and Meta-Analysis of Quality Improvement Interventions in OECD Countries
Source: PLoS One. 2013 Dec 19;8(12):e84464. doi: 10.1371/journal.pone.0084464 (PMC3868600; doi:10.1371/journal.pone.0084464)
Supplement: Table S5 — Characteristics and effectiveness of the interventions targeted to health system. QI= quality improvement; N= number of participants; CO = clinical outcomes; DSM = diabetes self-management; PC= processes of care; DM2= type 2 diabetes mellitus; RCT = randomized, controlled trial; BMI = body mass index; QE = quasi-experimental study; LDL-c= low-density lipoprotein cholesterol; HDL-c = high-density lipoprotein cholesterol; NA= not analyzed; HbA1c= glycated hemoglobin. *. Outcomes measures which showed a statistically significant improvement after the intervention are marked bold. (DOCX) [file pone.0084464.s005.docx]

Table S5. Characteristics and effectiveness of the interventions targeted to health system

| **QI strategy / Duration (months)** | **Setting of intervention** | **Provider of intervention** | **Target population** | **Components of the intervention** | **Design/ N/ Follow-up** | **Analyzed variables and main results*** | **Overall quality** | **Impact on CO** | **Impact on PC** | **Impact on DSM** | **Author (s)/ Country** |
| --- | --- | --- | --- | --- | --- | --- | --- | --- | --- | --- | --- |
| Electronic patient registry/38 | 24 primary care practices in Montana and 16 primary care practices in Wyoming which were communicated with patients’ homes | Health care professionals | Patients with DM2 living in medically underserved rural areas | Installation of software in the physician’s computer to monitor the health status of patients, and identify subgroups of patients needing special services. | QE (non-controlled before-after study)/ 37 Primary Care Practices participate (Montana=24, Wyoming=16), each state analyzed independently/ Follow up at completion | CO: Montana: **HbA_1c_/** Wyoming: HbA_1c._/ PC: Montana**: HbA_1c_ test in the past year, LDL-C test in the past year, foot examination in the past year, dilated retinal examination in the past year, pneumococcal vaccination ever received/** Wyoming: HbA_1c_ test in the past year, LDL-C test in the past year, foot examination in the past year, dilated retinal examination in the past year, **pneumococcal vaccination ever received.** | Poor | Partial | Partial | NA | Johnson et al. (2005)[41]/ United States |
| Electronic patient registry/24 | Federally Qualified Health Centers in West Virginia | Research team of the University of Virginia | Patients with DM2 from 6 Federally Qualified Health Centers in rural West Virginia | Implementation of an electronic patient registry. | QE (non-controlled before-after study)/ 6 Federally Qualified Health Centers participate (661providers)/ Follow up at completion | CO: **HbA_1c_, LDL-c**, HDL-c, **total** **cholesterol**, triglycerides, blood pressure /PC: **annual exams, screens to promote wellness, education, and self-management goal-setting** | Poor | Partial | High | NA | Pollard et al. (2009)[49] / United States |
| Case management + facilitated relay of information to clinicians/60 | Health centers of federally designated medically underserved areas in New York which were communicated with patient’ homes | Health professionals of primary care (family physicians, internal physicians, nurses and physician assistants) | Patients with DM2; age ≥55; residence in a federally designated medically underserved area in New York State. | Installation of a telemedicine system in patient’s homes, with 4 basic functions: 1. Videoconference service to interact with health professionals. 2. Remote monitoring of blood sugar and blood pressure. 3. Access to a portal to communicate with nurses. 4. Access to the American Diabetes Association website. | RCT/ N=1665 (intervention=844, control=821)/ Control group received usual care /Follow-up at 12 months post-baseline. | CO: **HbA1c, blood pressure, and LDL-c** | Good | High | NA | NA | Shea et al. (2006) [50]/ United States |
| Case management + facilitated relay of information to clinicians/60 | Health centers of federally designated medically underserved areas in New York which were communicated with patient’ homes | Health professionals of primary care (family physicians, internal physicians, nurses and physician assistants) | Patients with DM2; age ≥55; residence in a federally designated medically underserved area in New York State. | Installation of a telemedicine system in patient’s homes, with 4 basic functions: 1. Videoconference service to interact with health professionals. 2. Remote monitoring of blood sugar and blood pressure. 3. Access to a portal to communicate with nurses. 4. Access to the American Diabetes Association website. | RCT/ N=1665 (intervention=844, control=821)/ Control group received usual care /Follow-up at 12 months post-baseline. | DSM: **self-efficacy** | Good | NA | NA | High | Trief et al. (2007)[54]/ United States |
| Case management + facilitated relay of information to clinicians/60 | Health centers of federally designated medically underserved areas in New York which were communicated with patient’ homes | Health professionals of primary care (family physicians, internal physicians, nurses and physician assistants) | Patients with DM2; age ≥55; residence in a federally designated medically underserved area in New York State. | Installation of a telemedicine system in patient’s homes, with 4 basic functions: 1. Videoconference service to interact with health professionals. 2. Remote monitoring of blood sugar and blood pressure. 3. Access to a portal to communicate with nurses. 4. Access to the American Diabetes Association website. | RCT/ N=1665 (intervention=844, control=821)/ Control group received usual care./ Follow-up every 12 months till completion. | CO: **HbA1c, blood pressure, and LDL-c** | Good | High | NA | NA | Shea et al. [51] (2009) / United States |
| Case management + facilitated relay of information to clinicians/60 | Health centers of federally designated medically underserved areas in New York which were communicated with patient’ homes | Health professionals of primary care (family physicians, internal physicians, nurses and physician assistants) | Patients with DM2; age ≥55; residence in a federally designated medically underserved area in New York State. | Installation of a telemedicine system in patient’s homes, with 4 basic functions: 1. Videoconference service to interact with health professionals. 2. Remote monitoring of blood sugar and blood pressure. 3. Access to a portal to communicate with nurses. 4. Access to the American Diabetes Association website. | RCT/ N= 890 (Intervention = 447, Control = 443). Control group receives usual care /Follow-up at 12 and 24 months post-baseline | CO: BMI, waist circumference **/**DSM: **Exercise, Knowledge about diet** | Fair | Low | NA | High | Izquierdo et al. (2010) [40]/United States |
| Case management + facilitated relay of information to clinicians/60 | Health centers of federally designated medically underserved areas in New York which were communicated with patient’ homes | Health professionals of primary care (family physicians, internal physicians, nurses and physician assistants) | Patients with DM2; age ≥55; residence in a federally designated medically underserved area in New York State. | Installation of a telemedicine system in patient’s homes, with 4 basic functions: 1. Videoconference service to interact with health professionals. 2. Remote monitoring of blood sugar and blood pressure. 3. Access to a portal to communicate with nurses. 4. Access to the American Diabetes Association website. | RCT/ N=1665/ Whites (n= 821) Blacks (n= 248) Hispanic (585). Control group receives usual care/ Follow-up every 12 months till completion. | CO: HbA1c (white)/ CO:**HbA1c (black)/** CO:**HbA1c (Hispanic)** | Fair | High | NA | NA | Weinstock et al. (2011) [56]/United States |
| Case management + facilitated relay of information to clinicians/60 | Health centers of federally designated medically underserved areas in New York which were communicated with patient’ homes | Health professionals of primary care (family physicians, internal physicians, nurses and physician assistants) | Patients with DM2; age ≥55; residence in a federally designated medically underserved area in New York State. | Installation of a telemedicine system in patient’s homes, with 4 basic functions: 1. Videoconference service to interact with health professionals. 2. Remote monitoring of blood sugar and blood pressure. 3. Access to a portal to communicate with nurses. 4. Access to the American Diabetes Association website. | RCT/ N=1650 (intervention=837, control=813)/ Control group receives usual care/ Follow-up every 12 months till completion. | DSM: **Physical activity** | Good | NA | NA | High | Weinstock et al. (2011) [55]/United States |
