# Supplementary material for: Improving Diabetes Care in Rural Areas: A Systematic Review and Meta-Analysis of Quality Improvement Interventions in OECD Countries
Source: PLoS One. 2013 Dec 19;8(12):e84464. doi: 10.1371/journal.pone.0084464 (PMC3868600; doi:10.1371/journal.pone.0084464)
Supplement: Table S6 — Characteristics and effectiveness of multi-target interventions. QI= quality improvement; N= number of participants; CO = clinical outcomes; DSM = diabetes self-management; PC= Processes of care; DM2= type 2 diabetes mellitus; RCT = randomized, controlled trial; BMI = body mass index; QE = quasi-experimental study; LDL-c= low-density lipoprotein cholesterol; HDL-c = high-density lipoprotein cholesterol; NA= not analyzed; HbA1c= glycated hemoglobin; SMBG= self-monitoring blood glucose. *. Outcomes measures which showed a statistically significant improvement after the intervention are marked bold. (DOCX) [file pone.0084464.s006.docx]

Table S6. Characteristics and effectiveness of multi-target interventions

| **Target/ QI strategy / Duration (months)** | **Setting of intervention** | **Provider of intervention** | **Target population** | **Characteristics of the strategy/ies targeting patients** | **Characteristics of the strategy/ies targeting providers** | **Characteristics of the strategy/ies targeting the health system** | **Design/ N/ Follow-up** | **Analyzed variables and main results*** | **Overall quality** | **Impact on CO** | **Impact on DSM** | **Impact on PC** | **Author (s)/ Country** |
| --- | --- | --- | --- | --- | --- | --- | --- | --- | --- | --- | --- | --- | --- |
| **Patients** (Patient education + Promotion of self-management) + **Healthcare providers** (Audit and feedback + Clinician education)/24 | Primary care centers of a rural county in Indianapolis | Primary care physician, nurses, specialists in diabetes | Seven primary care physicians (all men, mean number of years in practice =21). Caucasian patients with DM2, living in a rural area in Indianapolis | **Patient education.** Several sessions of education about specific topics related to DSM were held for patients and their families. **Promotion of self-management.** A computer system was installed in the local hospital that enabled patients in constructing meal plans. | **Audit and feedback** Initially, the physician group developed consensus guidelines and agreed to adopt them. Chart audits were done on a randomly selected group of patients with diabetes, covering the year before the adoption of the guidelines. Information was provided to each physician about his own performance in terms of adherence to the guidelines, as well as information about performance of the group as a whole. **Clinician education.** A series of targeted sessions were held with primary care providers, covering various topics related to the guidelines. | NONE | QE (non-controlled before-after study)/ N=275/ Follow-up at completion | PC: **Blood pressure measured at each visit**, annual eye exam, annual referral to eye specialist, **annual comprehensive foot exam**, two or more HbA_1c_ measurements per year, annual lipid profile, annual protein or microalbumin test, insulin-treated patients doing self-monitoring of blood glucose, annual quitting advice to smokers | Fair | NA | NA | Partial | Kirkman et al. (2002) [43]/ United States |
| **Patients**(Patient education) + **Health System** (Case management)/5 | Family practice office in rural South Carolina | Dietitian and nurse case manager | Rural African American patients with DM2 living in a rural area in North Carolina | **Education of patients.** A. Dietary self-management education: 2 weekly group education 1-hour sessions about planning and preparing healthy food. B. Discussion groups: 1 30 minutes/1-hour monthly session. | NONE | **Case management** The nurse case manager provided follow-up through telephone and home visits to identify any complications, assist in solving problems, and help in making informed choices. Key finding were communicated to a physician liaison to improve care coordination and provide early intervention. | QE (non-controlled before-after study)/ N=23/ Follow-up at completion | CO: **HbA_1c_**, BMI, blood pressure, **glycaemia**, triglycerides, cholesterol/ DSM: **dietary habits** | Poor | Partial | High | NA | Anderson-Loftin et al. (2002) [32]/ United States |
| **Patients**  (Patient education + Reminder system) + **Health system (**case management + electronic patient registry) / 12 | 2 rural clinics in North Carolina | Health professionals of the health center where the intervention took place | Rural African American patients with DM2 from 2 primary care practices in 2 adjacent rural counties in eastern North Carolina | **Patient education.** Group sessions on nutrition, medication and self-management of diabetes. **Reminder system**. Implementation of a reminder program for patients to attend appointments, and recalls in case they miss them. | NONE | **Case management.** A nurse case manager was assigned to manage de control of diabetic patients. **Electronic patient registry.** Implementation of an electronic registry with the clinical characteristics of patients. | QE (controlled before-after study)/ N=160 (Intervention=112, Control=48). Control group: usual care/ Follow-up at completion | CO: **HbA_1c_**, weight, and blood pressure. | Fair | Partial | NA | NA | Bray et al. (2005) [36]/ United States |
| **Patients** (Patient education) + **Healthcare providers** (clinician education) + **Health system** (electronic patient registry + team changes)/ 28 | 3 primary care clinics in Montana | Health professionals of the health center where the intervention took place | Rural Patients with DM2 | **Patient education.** Six foot care clinics for patients with diabetes, implementation of a library of education materials, and development and dissemination of a newsletter that describes current patients, diabetes-related activities in the community and highlighted key aspects of diabetes self-management. | **Clinician education** Four members of the educational team, completed training in diabetes management and motivational techniques of counseling. | **Electronic patient registry:** Establishing and maintaining the patient registries. **Team changes:** nurses conducting mail and telephone outreach to patients in need of services (e.g., patients with elevated A1c values); mailing personalized patient education materials regarding the ABCs of diabetes (A1c, blood pressure, and low-density lipoprotein cholesterol [LDL-C] values) | QE (non-controlled before-after study)/ N= 213. /Follow-up at completion | PC: Blood pressure screening; **HbA1c test; LDL-C; Foot examination; Influenza immunization; Pneumococcal immunization (ever), Ever taken a class on managing diabetes; Ever taught SMBG by health Professional; SMBG at least weekly; Ever taught how to take care of your feet by health professional;** Treatment plan not working, Satisfaction with diabetes care/ CO: **HbA_1c_, Systolic blood Pressure, Diastolic blood Pressure,** LDL-c/ DSM: **Diabetes knowledge, Difficulty making lifestyle changes**; | Poor | High | High | Partial | Dettori et al. (2005)[39]/ United States |
| **Patients** (Patient education)+ **Healthcare providers** (clinician education)/12 | Family medicine healthcare center in the health centers network of the University of Pittsburgh | Diabetes educator and dietitian | Patients with DM2, age>18. Providers of a rural primary care practice (4 physicians, 1 nurse practitioner, and 1 physician assistant) | **Patient education.** 5 2-hour sessions of group education (5-10 persons) twice a week. The program consisted in changing behavior strategies. The educator monitored patients through telephone calls every 6 weeks. | **Clinician education.** Training of health professionals consisting in reviewing the recommendations of the American Diabetes Association, and emphasizing the importance of adherence to those guidelines. | NONE | QE (non-controlled before-after study)/ N=29/ Follow-up at completion | CO: **HbA_1c_,** LDL-c, **HDL-c**, blood pressure/ DSM: **patient empowerment, knowledge of diabetes/** PC: **number of HbA_1c_ measurements, lipid profiles, urinalysis, dilated eye exams and foot exams** | Poor | Partial | High | High | Siminerio et al. (2005) [52]/ United States |
| **Patients (**Patient education) + **Health system** (Case management) /6 | Primary care practice in rural South Carolina | Dietitian and nurse | African American patients with DM2 from 3 primary care practice sites; age≥18 | **Patient education.** A. Dietary self-management education: the main contents are planning, buying and preparing healthy food. 4 weekly sessions of 1.5 hours taught by a dietitian. B. Discussion groups: 5 monthly sessions of 1 hour by an African American nurse. Group dynamics and questions addressed at the nurse. | NONE | **Case management.** Weekly monitoring telephone calls conducted by the nurse for additional support, early identification of complications and problem solving. | RCT/ N=97 (intervention=49, control=48).Control group: Usual care/ Follow-up at completion | CO: HbA_1c_, lipidemia, **BMI/** DSM: **Dietary habits** | Fair | Partial | High | NA | Anderson-Loftin et al. (2005) [31] / United States |
| **Patients (**Patient education + Reminder system) + **Health System (**Case management + Electronic patient registry)/12 | 5 solo or small group primary care practices in rural North Carolina. | Nurse case manager, who visited each practice weekly for 12 months | Predominantly African American patients with DM2 from five rural clinics in North Carolina | **Patient education:** Group visits were scheduled in order to deliver focused educational content and material to a group of up to 10 diabetic patients from the same practice at the same time. **Reminder system:** The case manager also facilitated a return visit schedule and worked with office staff to create a patient reminder system as well as a call-back system for patients who missed appointments. | NONE | **Case management:** Intensive diabetes case management, which included revision of patients´ individual health plan and provided individual diabetes care. Also included facilitating laboratory testing as well as referrals to other providers. **Electronic patient registry:** System that allows office staff to enter demographic information regarding each patient with diabetes mellitus into a clinic population registry. Through a series of queries, detailed patient and laboratory information is available to the nurse case manager and physician. | QE (controlled before-after study)/ N= 314/ Follow up at completion | PC: **Self-management goal establishment, Lipid panel, Aspirin use, Foot examination** | Poor | NA | NA | High | Bray et al. (2005) [35]/ United States |
